# Synergistic O^2-^/Li^+^ Dual Ion Transportation at Atomic Scale

**DOI:** 10.34133/2019/9087386

**Published:** 2019-01-03

**Authors:** F. Q. Meng, Q. H. Zhang, A. Gao, X. Z. Liu, J. N. Zhang, S. Y. Peng, X. Lu, L. Gu, H. Li

**Affiliations:** ^1^Beijing National Laboratory for Condensed Matter Physics, Institute of Physics, Chinese Academy of Sciences, Beijing 100190, China; ^2^School of Materials, Sun Yat-Sen University, Guangzhou 510275, China; ^3^College of Materials Science and Engineering, Jiangxi University of Science and Technology, Jiangxi 341000, China; ^4^Songshan Lake Materials Laboratory, Dongguan, Guangdong 523808, China; ^5^School of Physical Sciences, University of Chinese Academy of Sciences, Beijing 100049, China

## Abstract

The ion migration during electrochemical process is a fundamental scientific issue for phase transition behavior and of technical importance for various functional devices, where cations or anions are active under electrical bias. Usually only one type of functional ion, O^2-^ or Li^+^, is activated due to their different migration energy barriers, cooperated by the valence change of other immobile ions in the host lattice matrix, e.g., Co^2+^/Co^3+^ and Mn^3+^/Mn^4+^ redox couples, owing to the charge neutralization. Here we select spinel Li_4_Ti_5_O_12_ as anode and construct an all-solid-state battery under a transmission electron microscope; a synergistic transportation of O^2-^ and Li^+^ driven by an electrical bias was directly observed at the atomic scale. A small amount of oxygen anions was extracted firstly as a result of its lowest vacancy formation energy under 2.2 V, leading to the vertical displacement of oxygen. Up to 2.7 V, an ordered phase with both Li- and O- deficiency formed. The Li^+^ and O^2-^ ions are simultaneously extracted out from the [LiO_4_] tetrahedra due to the electroneutrality principle. The migration paths of O and Li have been proposed and verified by first-principles calculations. These results reveal a brand new synergistic ion migration manner and may provide up-to-date insights on the transportation process of lithium ion conductors.

## 1. Introduction

Electrochemical phase transition involves various migration processes of ions, which is the foundation of its functionality, especially in the operation of lithium-ion battery (LIB) materials where the Li ions shuffle between anode and cathode during charge-discharge process [1–3]. Usually only one type of functional ions responses to the external electric field, cooperated by the valence change of other immobile ions in the host lattice matrix due to the charge neutralization. For example, the extraction and insertion of lithium are accompanied by the oxidation and reduction of the transition metal ions, Co^2+^/Co^3+^ and Mn^3+^/Mn^4+^ redox couples, in typical anode materials LiCoO_2_ and LiMn_2_O_4_, respectively [4, 5]. However, the situation of lithium extraction without valence-changeable cations, where the charge compensation cannot be accomplished by the oxidation of transition metal ions, is a less investigated area but deserves deep understandings. At this point, we demonstrate the detailed migration process of Li^+^ and O^2-^ when we further extracted the lithium from the stable spinel Li_4_Ti_5_O_12_ (LTO) with the chemically stabilized Ti^4+^ ions upon delithiation.

**Figure 1:**
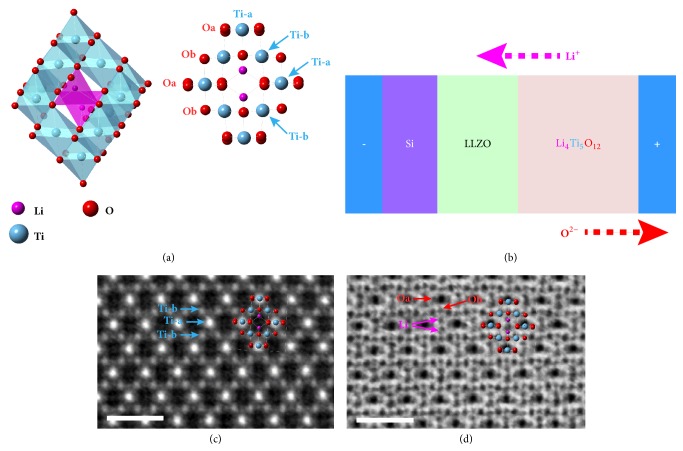
**Atomic structure of Li**_**4**_ **Ti**_**5**_ **O**_**12**_ **and its in-situ experiment setup. **(a) Structural schematics of Li_4_Ti_5_O_12_ and the projection along the <110> direction. Ti columns with different projection density are labeled with Ti-a and Ti-b, the neighbored oxygen columns are also labeled with Oa and Ob, respectively. (b) In-situ setup for constructed batteries where Li_4_Ti_5_O_12_ serves as positive electrode, the LLZO acts as the electrolyte and Si as the counter-electrode. (c, d) HAADF and ABF images of Li_4_Ti_5_O_12_ viewed along the <110> direction, the Ti, O and Li columns have been identified at atomic scale. Scale bar: 1 nm.

Crystallographically, LTO spinel structure can be expressed as [Li]_8a_[Li_1/3_Ti_5/3_]_16d_[O_4_]_32e_ as displayed in Figure 1(a), where one Li^+^ ion occupies the 8*a* sites with tetrahedral coordination, the remaining 1/3 Li^+^ and all Ti^4+^ ions being in the 16*d* sites with octahedral coordination and the O^2-^ ions in the 32*e* sites. The [TiO_6_] octahedra share faces with the [LiO_4_] tetrahedra. There are also empty tetrahedral 8*a* and octahedral 16*c* sites interconnected by edge-sharing octahedral sites, providing the interstitial space for the ion migration. In fact, the Li_4_Ti_5_O_12_ is mostly used as an anode material in lithium ion batteries (LIBs) [6]. During lithiation, the Coulombic interaction between tetrahedral sites drives the migration of Li^+^ ions from the tetrahedral 8*a* site to the empty octahedral 16*c* sites with a small energy barrier ~ 0.3 eV, resulting in the Li_7_Ti_5_O_12_ phase with rock-salt structure in two-phase separation mechanism [7–9] and the partial reduction of Ti^4+^ to Ti^3+^ ions [7, 10–12]. During the delithiation, the reverse ion migration process and phase transition from the rock-salt Li_7_Ti_5_O_12_ to the spinel Li_4_Ti_5_O_12_ occur, accompanying the oxidation of partial Ti^3+^ to tetravalent Ti ions. However, in practical operation of LIBs with the Li_4_Ti_5_O_12_ anode, the over-discharging is always unavoidable, which is directly correlating with the capacity fading upon cycling. The charge compensation for the further extraction of Li ions from the Li^+^_4_[Ti^4+^]_5_O^2−^_12_ seems significantly difficult from the Ti^4+^ ions due to its stabilized electron configuration, thus promising that from the lattice oxygen ions upon the Li extraction. However, it is unclear whether the oxygen ions would be oxidized to the O^−^ state or escaped from the Li_4_Ti_5_O_12_ lattice as the O_2_ gas. Therefore, it is desirable to reveal the structure evolution under the electric extraction of Li ions to understand the degradation mechanism of Li_4_Ti_5_O_12_ spinel.

## 2. Results

### 2.1. In Situ Setup for Ion Extraction

We constructed an all-solid-sate battery in the TEM by a newly developed chip-based* in situ* technique with focused ion beam (FIB) milling [13], which allows the atomic resolution imaging under electric bias, and investigated the structural evolution of Li_4_Ti_5_O_12_ during the lithium extraction. As shown in Figure 1(b), we used the LLZO as the electrolyte and Si as the counterelectrode and applied a positive bias on the Li_4_Ti_5_O_12_ electrode. Figure 1(c) demonstrates the high-angle dark-field (HAADF) image of Li_4_Ti_5_O_12_ viewed along the [110] direction under this electrical configuration, where Ti columns are imaged as the strong white spots (Ti-a arrays) and weak white spots (Ti-b arrays) because the Ti content in Ti-a columns is roughly twice that in the Ti-b columns as indicated by red arrows, while the O and Li are hardly visualized due to the Z^2^ dependence of HAADF contrast (Z is elemental number). On the other hand, the angular bright-field (ABF) imaging geometry is extremely feasible to image light elements such as lithium, where the image contrast is of ~Z^1/3^ dependence [14, 15]. Consequently, the Li and O columns can be observed clearly in the ABF image as displayed in Figure 1(d). Here we define the oxygen in the Ti-a array as Oa and that in the Ti-b arrays as Ob. We can see that the vertical atomic displacement in the Oa arrays is more prominent than that in the Ob arrays within the Ti (001) plane, which is in good agreement with the pristine structure shown in Figure 1(a). It should be noted that although the Z of Li is smaller than that of O, the ABF contrast of Li shown here is comparable to that of O due to the thickness effect [16].

### 2.2. Formation of Oxygen Vacancies and Related Atomic Shift at 2.2 V

**Figure 2:**
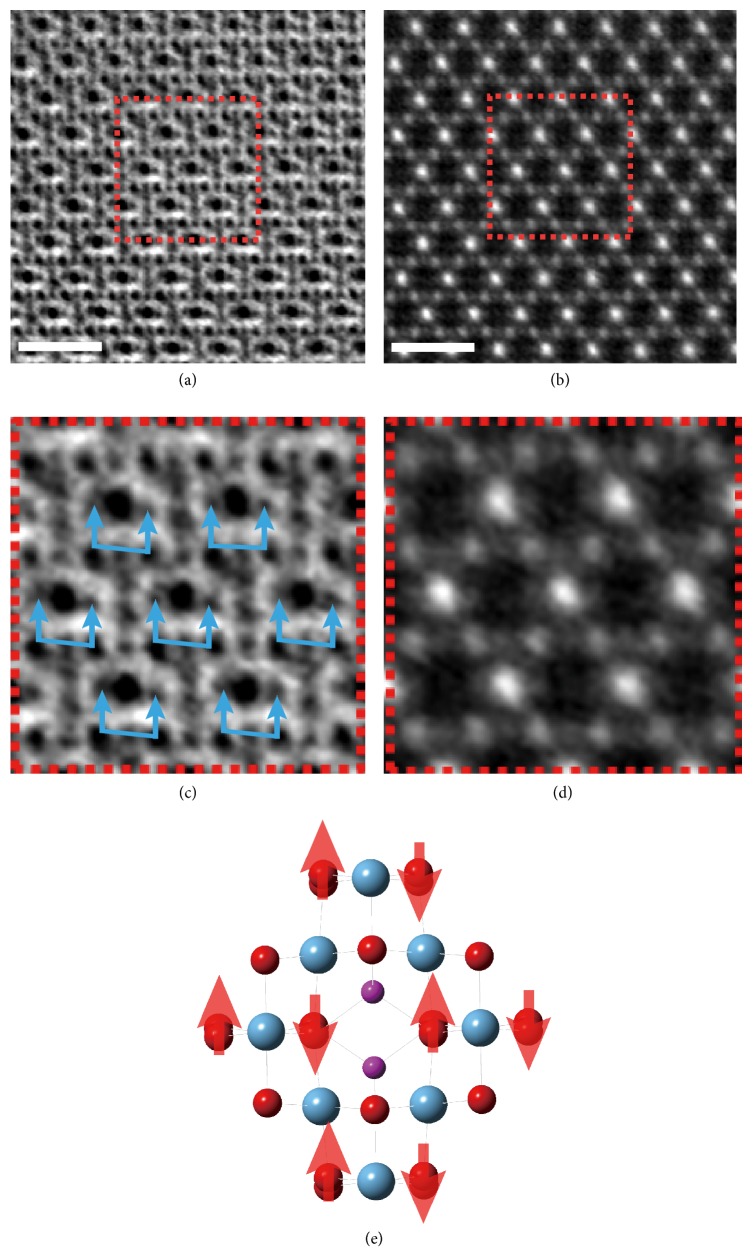
**STEM images of Li**_**4-x1**_ **Ti**_**5**_ **O**_**12-y1**_ **under 2.2 V. **(a, b) HAADF and ABF images under 2.2 V, respectively. (c, d) the enlarged HAADF and ABF images from the red dotted frame in (a, b). Blue arrows in ABF image (c) indicated the oxygen atomic shift. Schematics of oxygen atomic shift (e), brown and purple arrows are used to label the displacement direction in the Ti-a and Ti-b columns, respectively. Scale bar: 1 nm.

When an appropriate positive bias was applied on the top electrode, in LTO electrode the Li^+^ ions would move towards LLZO and O^2-^ ions towards top electrode, as indicated by arrows in Figure 1(b). Then the voltage was increased from the 0 V to 2.2 V, where little change was detected from the Ti diamond of the spinel lattice as shown in the HAADF image (Figures 2(b) and 2(d)), while the vertical displacement of oxygen at the Oa sites becomes more obvious with respect to the vertical displacement in the Ob arrays decrease and lead to a nearly flat arrangement (Figures 2(a) and 2(c)) as depicted in the ABF images. We schematize this change in Figure 2(e), where the purple and dark-yellow arrows indicate the vertical displacement of the Oa and the Ob arrays under 2.2V, with reference to the pristine Li_4_Ti_5_O_12_ lattice. This atom transfer of oxygen may be induced by the presence of some oxygen vacancies—interstitial oxygen cluster as reported by Kanno et al. [17]. Meanwhile, the Li ions at 8*a* sites slightly shift away from their original positions due to the presence of O vacancies, similar to the situation in defected spinel Li_1−x_Mn_2_O_4-*δ*_ [18]. Thus, the atomic displacement of oxygen indicates the forever n of a certain amount of oxygen vacancies under a modest (2.2 V) electric bias.

To find out which ion is more likely to be extracted firstly, we compared the vacancy formation energy at the Li- and O- sites in spinel Li_4_Ti_5_O_12_ by first-principles calculations (simulation details refer to our previous work [7, 9, 19]). The results are 4.88 eV, 5.10 eV, and 4.62 eV for the Li-Vacancy (V_Li_) at 8*a* sites, V_Li_ at 16*d* sites, and O-Vacancy (V_O_) at 32*e* sites. Thus, the V_O_ would be created firstly under electric bias due to its smallest formation energy, while the Li removal (8*a* site) will almost be accompanied with the O loss. This is in agreement with our observation of oxygen shift under 2.2 V due to the oxygen defects. Thus, the chemical formula of the defected spinel LTO can be depicted as Li_4-*x*_Ti_5_O_12-*δ*_. Here the* x* indicates that some Li ions also should be extracted out from the lattice due to the charge compensation mechanism.

### 2.3. Li Extraction and Oxygen Vacancy Ordering at 2.7V

**Figure 3:**
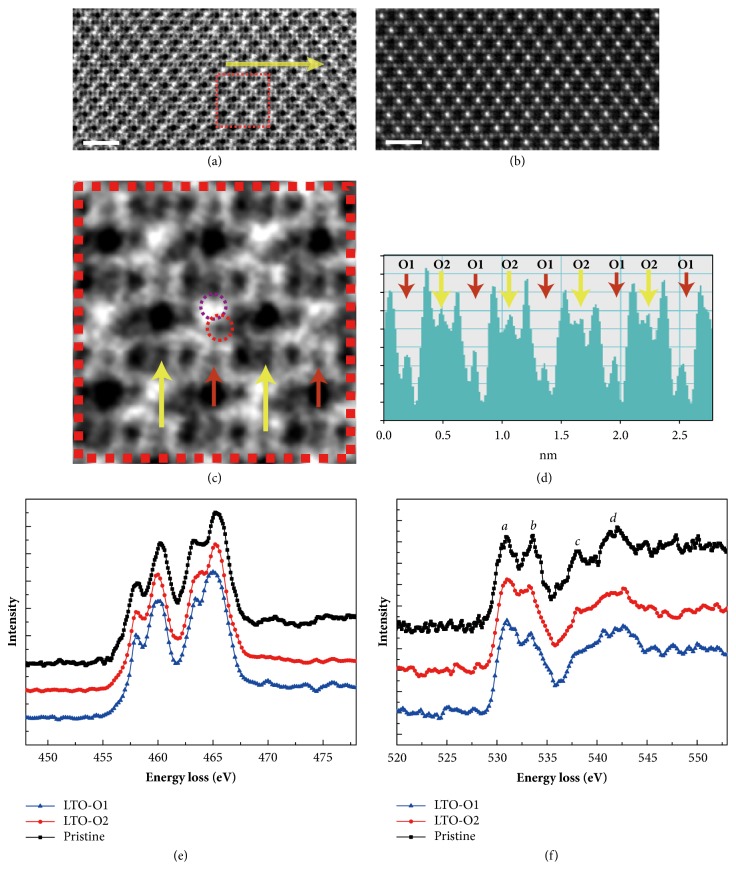
**STEM images and EEL spectra of Li**_**4-x2**_ **Ti**_**5**_ **O**_**12-y2**_ **under 2.7 V.** (a) ABF and (b) HAADF images of the oxygen-vacancy ordered Li-deficient LTO. (c) Enlarged ABF image from the red frame in (a). (d) Line profile of inversed ABF contrast along the [Ti-b]-[Ob]-[Ti-b] chain. (e) Ti L edges and (f) O K edge of O1 sites and O2 sites, which are normalized by peak* a*. Scale bar: 1 nm.

An obvious structural change emerges at 2.7V. As shown in Figure 3(a), a bright contrast in the inner of the Ti diamond appears in the ABF image, indicating lots of vacancies in the 8*a*-Li sites. From the enlarged ABF image in Figure 3(c), we find that the contrast of the Li ions at the upper 8a sites (outlined by a small green dotted circle) disappears, indicating the partial extraction of Li ions from the defected spinel under 2.7 V. The rest of the Li ions deviated from the lower 8a site and shifted towards one of the Oa sites (outlined by a small red dotted circle). Meanwhile, the oxygen at Ob sites also exhibits periodic change in ABF intensity as indicated by the yellow and small brown arrows in Figure 3(c), where an obvious decision decrease of oxygen occupation can be visualized in the Ob sites labeled by small brown arrows. Line profile of the ABF image contrast along the [Ti-b]-[Ob]-[Ti-b] chain (Figure 3(d)) from the positions indicated by the yellow arrows in Figure 3(a) also demonstrated an obvious periodic intensity modulation (yellow and small brown arrows) at the Ob sites, which reflects the occupancy variation of oxygen and formation of ordered oxygen vacancies. For clarity, O1 and O2 are used to distinguish these two types of Ob sites as labeled in Figure 3(d). It means that accompanying the Li extraction from one of the 8*a* sites, the oxygen in one of the Ob sites simultaneously migrated out to satisfy the charge compensation and resulted in an oxygen-vacancy ordered Li-deficient structure when the amount of the oxygen vacancy reached a threshold. Line scan EEL spectra have also been acquired along the [Ti-b]-[Ob]-[Ti-b] chain and compared with the pristine state of LTO at two distinct oxygen sites O1 and O2 can be identified. No obvious changes in the Ti L edges were observed, indicating the valence state invariance of Ti^4+^, as shown in Figure 3(e), while obvious decrease of peaks* b*,* c*, and* d* in the O K edges can be visualized in Figure 3(f) which manifests the presence of oxygen vacancies [20]. More importantly, the decrease of* b*,* c*, and* d* peak at the O1 sites is more prominent than that at the O2 sites, which is in good agreement with the modulation of oxygen content shown in ABF image (Figure 3(a)).

## 3. Discussion

### 3.1. Discussion on Ion Migration Path

**Figure 4:**
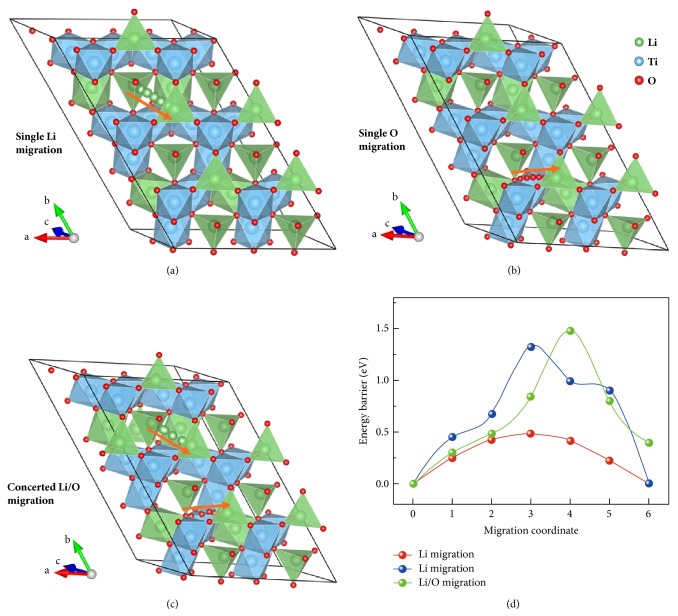
**Theoretical calculations on ion diffusion.** The Li (a), O (b) Li-O (c) concerted diffusion inside a 1×3×1 LTO supercell with the corresponding activation energies (d).

Then a 1×3×1 LTO supercell was built to simulate the Li and O transport features (details on the structural construction and Li-Ti 16d antisite optimization refer to our previous work [14]).  Figure 4 shows the Li, O, and Li-O concerted diffusion inside the LTO supercell, respectively. Upon the formation of O vacancy, the simulation results demonstrated that one V_O_ introduces obvious lattice distortion, leading to one five-coordinated [LiO_5_] polyhedra surrounded by three [TiO_6_] octahedra, by which the local structural distortion was generated to modulate the Li and/or O migration behavior [9]. From the STEM images as shown in Figures 2 and 3, the contrast decrease in O columns becomes increasingly obvious, indicating that the O ions inside the LTO spinel are gradually extracted out at a higher electric bias. From a thermodynamic point of view, the mobile O ions would probably interact with each other to release the O_2_ gas. At the same time, the delithiation process occurs to establish the charge balance. In this respect, the extraction of Li and O ions in LTO spinel could possibly happen in a synergetic way.

Figures 4(a) and 4(b) manifest the single Li or O ions diffusion inside the LTO spinel with their activation energy shown in Figure 4(d). Upon a Li ion migration, the standard 8*a*-16*c*-8*a* 3D diffusion feature is derived from simulations. The activation energy of Li ion diffusion is estimated to be 0.48 eV, while the one step hopping of O ions needs to overcome an energy barrier of 1.33 eV, indicating the mobile possibility at such higher electric bias. Then if the synergetic migration of Li and O ions inside the LTO spinel is considered, the total energy barrier is approx. 1.48 eV, a little higher than the single O ion migration, which means the possible interaction between the Li and O ions during the dynamic transport process. This also accounts for the STEM observations in Figures 3 and 4. Further simulation results indicated that there is a higher activation energy for the Li-O dual ion migration process if the Li and O trajectories are very close with each other and the diffusion trajectory will be much more intricate as well.

Similar synergistic transport behavior of dual Li and O ions could be extended to other functional oxides without valence-changeable cations, where extra capacity can be expected due to the extra Li extraction. Contrary to O2 gas release process in other Li- and Mn-rich cathode materials [21], the oxygen loss here does not introduce obvious reduction of Ti and structural degradation, as shown in Figures 3(b) and 3(e). In addition, a shrinking of 1.25% along the short diagonal and an expansion of 4.67% along the long diagonal of the Ti diamond were obtained from the HAADF images under 2.7 V, respectively. This relative low lattice strain should be related to the simultaneous extraction of dual Li and O ions where the electrostatic repulsive force due to the Li extraction between O^2-^ layers is cancelled to a certain extent, promising a good stability during the synergistic transport process.

### 3.2. Conclusion

The synergistic transport processes of O^2-^ and Li^+^ ions under electric field are observed directly at the atomic scale, and an ordered oxygen deficient phase was identified subsequently at a higher voltage. A small amount of oxygen anions was extracted firstly and led to the vertical displacement of oxygen under 2.2 V; an ordered phase with both Li- and O- deficiency formed up to 2.7 V. The migration path of Li^+^  and O^2-^ ions has been discussed by first-principle calculations. These results reveal that the Li^+^ and O^2-^ ions are simultaneously extracted out from the [LiO_4_] tetrahedral, demonstrating a new synergistic migration manner of functional ions in lithium ion conductors.

## 4. Materials and Methods

### 4.1. STEM Experiments

High-resolution STEM investigation was carried out on a JEM-ARM200F microscope with double C_S_ correctors for the condenser lens and objective lens. The available point resolution is better than 0.08 nm at an operating voltage of 200 kV. HAADF and ABF images were acquired at acceptance angles of 70-150 mrad and 11-22 mrad, respectively. All of the images presented here are Fourier-filtered to minimize the effect of the contrast noise. The filtering does not have any effect on the results of our measurements. In addition, we adjusted deliberately the brightness and contrast in order to better represent the atomic arrangements; this adjustment does not have any effect on the result.

### 4.2. DFT Calculations

Spin-polarized calculations were performed using the Vienna Ab Initio Simulation Package within the projector augmented-wave approach [22–24]. Generalized gradient approximation in the parameterization of Perdew, Burke, and Ernzerh was used to describe the exchange-correlation potential [25–27]. The cutoff of the kinetic energy was set to 600 eV for all calculations. Geometry optimization was performed using a 1*a∗*3*b∗*1*c* supercell and considered converged when the force on each atom was <1 meV Å^−1^. The Brillouin zone integration was performed with 3*∗*1*∗*3 and 1*∗*1*∗*1 Γ centered Monkhorst-Pack k-point meshes [28] in geometry optimization and climbing image nudged elastic band calculations, respectively.

## Data Availability

All data needed to evaluate the conclusions in the paper are present in the paper. Additional data related to this paper may be requested from the authors (QH Zhang: zqh@iphy.ac.cn).
